# Encorafenib with Binimetinib for the Treatment of Patients with BRAF V600 Mutation-Positive Unresectable or Metastatic Melanoma: An Evidence Review Group Perspective of a NICE Single Technology Appraisal

**DOI:** 10.1007/s41669-020-00206-x

**Published:** 2020-04-14

**Authors:** Rachel Houten, Janette Greenhalgh, James Mahon, Sarah Nevitt, Sophie Beale, Angela Boland, Tosin Lambe, Yenal Dundar, Eleanor Kotas, Joanne McEntee

**Affiliations:** 1 Liverpool Reviews and Implementation Group, University of Liverpool, Whelan Building, Liverpool, L69 3GB UK; 2 Coldingham Analytical Services, Berwick-upon-Tweed, UK; 3 North West Medicines Information Centre, Liverpool, L69 3GF UK

## Abstract

As part of the Single Technology Appraisal process, the National Institute for Health and Care Excellence (NICE) invited Pierre Fabre to submit evidence for the clinical and cost-effectiveness of encorafenib with binimetinib (Enco + Bini) versus dabrafenib with trametinib (Dab + Tram) as a first-line treatment for advanced (unresectable or metastatic) BRAF V600 mutation-positive melanoma. The Liverpool Reviews and Implementation Group at the University of Liverpool was commissioned as the Evidence Review Group (ERG). This article summarises the ERG’s review of the company’s evidence submission (CS), and the Appraisal Committee’s (AC’s) final decision. The main clinical evidence in the CS was derived from the COLUMBUS trial and focused on the efficacy of Enco + Bini (encorafenib 450 mg per day plus binimetinib 45 mg twice daily) compared to vemurafenib. The company conducted network meta-analyses (NMAs) to indirectly estimate the relative effects of progression-free survival (PFS), overall survival (OS), adverse events (AEs) and health-related quality of life (HRQoL) for Enco + Bini versus Dab + Tram. None of the results from the NMAs demonstrated a statistically significant difference between the treatment regimens for any outcomes. The ERG advised caution when interpreting the results from the company’s NMAs due to limitations relating to the methods. The ERG considered that use of the OS and PFS hazard ratios (HRs) generated by the company’s NMAs to model the relative effectiveness of Enco + Bini versus Dab + Tram in the company model was inappropriate as these estimates were not statistically significantly different. The ERG amended the company’s economic model to include estimates of equivalent efficacy, safety and HRQoL for Enco + Bini and Dab + Tram. The ERG considered use of different estimates of relative dose intensity to be inappropriate and used the same estimate for both drug combinations. The ERG also concluded that as only the prices of drug combinations were different, a cost comparison was an appropriate method of economic analysis. Using this approach (combined with confidential discounted drug prices for Enco + Bini and Dab + Tram), treatment with Enco + Bini was more cost effective than treatment with Dab + Tram. The AC raised concerns that an absence of evidence of a difference in outcomes between Enco + Bini and Dab + Tram did not constitute evidence of absence. However, as the numerical differences in outcomes generated by the company’s networks were small, the AC did not have a preferred approach and considered that both the company’s and the ERG’s methods of incorporating outcome estimates into the economic model were suitable for decision making. The NICE AC recommended Enco + Bini as a first-line treatment for unresectable or metastatic melanoma with a BRAF V600 mutation.

## Key Points for Decision Makers

**Table:** 

There is no direct evidence for the comparative effectiveness of encorafenib with binimetinib (Enco + Bini) and dabrafenib with trametinib (Dab + Tram), and the Evidence Review Group (ERG) considered the available indirect evidence was unreliable.
For patients with BRAF V600 mutation-positive unresectable or metastatic melanoma, there is no evidence comparing the effectiveness of targeted treatments with immunotherapies even though both are recommended by the National Institute for Health and Care Excellence (NICE) in this patient group.
The ERG presented a cost comparison (using confidential discounted drug prices) of Enco + Bini versus Dab + Tram that demonstrated that Enco + Bini was the more cost-effective option.
The NICE Appraisal Committee considered that treatment with Enco + Bini was likely to represent a cost-effective use of National Health Service (NHS) resources when compared to Dab + Tram.

## Introduction

The National Institute for Health and Care Excellence (NICE) is an independent organisation responsible for providing national guidance to the National Health Service (NHS) in England and Wales on a range of clinical and public health issues, including the appraisal of new health technologies. The NICE Single Technology Appraisal (STA) process is specifically designed for the appraisal of a single health technology for a single indication, where most of the relevant evidence lies with one manufacturer or sponsor and typically covers new technologies shortly after UK market authorisation is granted [1]. Within the STA process, the manufacturer or sponsor provides a written submission (alongside a decision analytic model) that summarises an estimate of the clinical and cost-effectiveness of the technology. An external independent organisation (typically, an academic group) known as the Evidence Review Group (ERG), provides a critique of the company’s submission (the ERG report). Consultees, clinical specialists and patient representatives also provide additional information during the appraisal process.

Using a specification developed by NICE (the final scope), the NICE Appraisal Committee (AC) considers the company’s submission [2], the ERG report, and testimonies from experts and stakeholders to determine whether the technology represents a clinically effective and cost-effective use of NHS resources. All stakeholders and the public have an opportunity to comment on the preliminary guidance issued by NICE in the form of an Appraisal Consultation Document (ACD), after which the AC meets again to produce the final guidance (Final Appraisal Determination [FAD]). The final guidance constitutes a legal obligation for NHS providers in England and Wales to provide a technology that is approved within its licensed indication [1].

This article presents a summary of the ERG report for the STA of encorafenib with binimetinib (Enco + Bini) as a treatment for patients with advanced (unresectable or metastatic) BRAF V600 mutation-positive melanoma. The Liverpool Reviews and Implementation Group at the University of Liverpool was commissioned to act as the ERG for this STA. Full details of all relevant appraisal documents (including the appraisal scope, ERG report, company and consultee submissions, NICE guidance, and comments on each of these) can be found on the NICE website [2].

## Decision Problem

Melanoma is the fifth most common cancer in the UK [3]. Incidence rates have increased in the last 30 years and are expected to increase by a further 7% by 2035 [4]. Prevalence amongst the younger age groups is higher in women, whilst more men in older age groups present with melanoma. Australia and New Zealand have the highest rates of melanoma in the world [5, 6].

In the UK, in 2016, almost 14,000 new cases of melanoma were diagnosed [3]. Approximately 9% of all melanoma patients are diagnosed with advanced melanoma (stage III or stage IV) [4], and approximately 20% of people treated for early stage primary melanoma progress to an advanced stage [7]. The 5-year survival rate for stage IIIB melanoma is around 60%, although the survival rate at 1-year for stage IV disease may be as low as 33% [4].

Almost half of all melanomas express a mutated form of the B-Raf proto-oncogene kinase (BRAF) and the majority of these are BRAF V600 mutations [8]. The specific patient population considered in this appraisal is patients with advanced (unresectable or metastatic) BRAF V600 mutation-positive melanoma. At the time of this appraisal (February 2019), the standard of care in the UK for this patient group was systemic treatment with either a targeted treatment or with an immunotherapy [9]. Targeted treatments include dabrafenib with trametinib (Dab + Tram) [10], or monotherapy with vemurafenib [11] or dabrafenib [12]. Immunotherapies include nivolumab with ipilimumab [13], nivolumab monotherapy [14, 15], pembrolizumab monotherapy [16, 17] and ipilimumab monotherapy [18]. There is no consensus as to whether targeted treatment should be used prior to immunotherapy or vice versa. Clinical advice to the ERG was that, in the NHS, immunotherapies are used as a first-line treatment in the majority of patients. Targeted therapies are generally only used as a first-line treatment in patients with a high disease burden.

Encorafenib is licensed in Europe [19] for use in combination with binimetinib, for the treatment of patients with BRAF V600 mutation-positive unresectable or metastatic melanoma. Encorafenib is available in 50-mg and 75-mg hard capsules. The recommended dosage of encorafenib, when used with binimetinib, is 450 mg (six 75-mg capsules) once daily. Binimetinib is available in 15-mg film-coated tablets. The recommended dosage of binimetinib, when used with encorafenib, is 45 mg (three 15-mg tablets) twice daily [19].

NICE developed a scope for the assessment of Enco + Bini, which specified that the clinical and cost-effectiveness of this combination treatment should be established within its licensed indication relative to Dab + Tram. Five measures of clinical effectiveness were considered relevant for this appraisal: overall survival (OS), progression-free survival (PFS), response rate, adverse events (AEs) and health-related quality of life (HRQoL). If sufficient evidence was available, then subgroup data was to be considered for people with previously untreated disease and for people with previously treated disease that had progressed on or after first-line immunotherapy.

## The Independent ERG Report

The company provided a submission to NICE describing the use of Enco + Bini (within the context of its licensed indication) in adults with advanced (unresectable or metastatic) BRAF V600 mutation-positive melanoma. The comparator for this appraisal was Dab + Tram [20]. The ERG examined and critiqued both the initial and subsequent evidence submissions from the company as well as taking into consideration the company’s response to their request for clarification on a number of issues. The ERG report comprised a critical review of the evidence of the clinical and cost-effectiveness of the technology based upon the company’s submissions to NICE. The review embodied three aims:

- To assess whether the company’s submission conformed to the methodological guidelines issued by NICE
- To assess whether the company’s interpretation and analysis of the evidence were appropriate
- To indicate the presence of other sources of evidence or alternative interpretations of the evidence that could help inform NICE guidance.

In addition to providing this detailed critique, the ERG modified a number of key assumptions and parameters within the company’s economic model to examine the impact of such changes. This section summarises the evidence submitted by the company and the ERG’s review of that evidence.

### Clinical Evidence

#### Direct Evidence

The company conducted a literature search that identified a single randomised controlled trial (RCT), the COLUMBUS trial [21–23]. The COLUMBUS trial [21–23] is an international, randomised, open-label, phase III trial designed to assess the clinical effectiveness of the licensed dose of Enco + Bini compared with vemurafenib and compared with encorafenib monotherapy (Enco) in 577 patients with advanced (unresectable or metastatic) BRAF V600 mutation-positive melanoma. The results for patients randomised to the Enco arm were not relevant to the present appraisal and will not be discussed in any detail here. The COLUMBUS trial [21–23] did not compare Enco + Bini with Dab + Tram, the comparator specified in NICE’s final scope.

The direct evidence from the COLUMBUS trial [21–23] is presented in Table 1. The primary objective of the COLUMBUS trial [21–23] was to compare PFS between Enco + Bini and vemurafenib based on blinded independent central review (BICR). At the data cut-off date of 19 May 2016, median PFS was 14.9 months (95% confidence interval [CI] 11.0–18.5) and 7.3 months (95% CI 5.6–8.2) in the Enco + Bini and vemurafenib arms, respectively. The difference was statistically significant in favour of Enco + Bini: hazard ratio (HR) 0.54 (95% CI 0.41–0.71); stratified one-sided log-rank test *p* < 0.0001.

**Table 1:** PFS and OS in the ITT population of the COLUMBUS trial

Outcome	Enco + Bini (*N* = 192)	Vemurafenib (*N* = 191)
PFS		
Number of events (%)	98 (51.0)	106 (55.5)
Median follow-up time in months (95% CI)	16.7 (16.3–18.4)	14.4 (10.1–16.6)
50th (median) percentile of PFS (95% CI)	14.9 (11.0–18.5)	7.3 (5.6–8.2)
HR (95% CI) and stratified one-sided log-rank *p* value	0.54 (0.41–0.71); *p* < 0.0001	
OS		
Median follow-up time in months (95% CI)	37.2 (36.1–38.5)	35.9 (34.9–38.0)
Median OS (95% CI)	33.6 (24.4–39.2)	16.9 (14.0–24.5)
HR (95% CI) and *p* value	0.61 (0.47–0.79); *p* < 0.0001	

*Bini* binimetinib, *CI* confidence interval, *Enco* encorafenib, *HR* hazard ratio, *ITT* intention to treat, *OS* overall survival, *PFS* progression-free survival

OS outcomes could not be formally tested, according to the pre-specified hierarchical approach of statistical testing, since Enco + Bini did not demonstrate a statistically significant advantage over Enco (HR 0.75; 95% CI 0.56–1.00). Nominal *p* values for OS from the interim OS analysis (7 November 2017) were, therefore, only descriptive. Median OS was 33.6 months (95% CI 24.4–39.2) in the Enco + Bini arm and 16.9 months (95% CI 14.0–24.5) in the vemurafenib arm. The HR for the comparison of Enco + Bini with vemurafenib was 0.61 (95% CI 0.47–0.79; nominal one-sided *p* < 0.0001). Results of updated, supportive and sensitivity analyses of primary and key secondary efficacy outcomes were consistent with the results of the primary analysis.

The HRQoL results from the COLUMBUS trial [21–23] demonstrated that Enco + Bini statistically significantly delayed deterioration in quality of life compared with vemurafenib, as measured by median time to 10% deterioration on the Functional Assessment of Cancer Therapy-Melanoma (FACT-M) subscale, the European Organisation for Research and Treatment of Cancer Quality of Life Questionnaire-Core 30 (EORTC-QLQ-C30) global health status and the European Quality of Life-5 Dimensions Questionnaire (EQ-5D-5L) questionnaire.

The frequencies of AEs were similar across the three arms of the COLUMBUS trial [21–23]. Patients treated with Enco + Bini had a longer time on treatment compared with patients treated with vemurafenib. The most frequently reported ≥ grade 3 serious AEs in ≥ 2% of patients treated with Enco + Bini were pyrexia and anaemia, and, in the vemurafenib arm, they were general physical health deterioration and back pain. The most common All-grade serious AEs (≥ 2.0% of patients) in the Enco + Bini arm were pyrexia, abdominal pain, acute kidney injury and anaemia; in the vemurafenib arm, the only common all grade serious AE was general physical health deterioration.

The ERG noted that the clinical efficacy outcomes and the HRQoL outcomes of the COLUMBUS trial [21–23] favoured the use of Enco + Bini and that Enco + Bini appeared to be well tolerated by patients.

#### Indirect Evidence

In the absence of direct evidence comparing Enco + Bini versus Dab + Tram, the company conducted Bayesian network meta-analyses (NMAs) to indirectly estimate the relative effects of treatment efficacy (PFS and OS), safety and HRQoL. The company identified seven RCTs (COLUMBUS [21–23], COMBI-v [24, 25], COMBI-d [26–28], BRF113220 Part C [29–33], coBRIM [34–36], BREAK-3 [31, 37, 38] and BRIM-3 [39–42]) that were designed to investigate the efficacy of BRAF inhibitor (BRAFi) therapies. Clinical efficacy and safety data were available from all of the trials, whilst HRQoL data were collected in five trials (COLUMBUS [21–23], COMBI-v [24, 25], COMBI-d [26–28], coBRIM [34–36] and BREAK-3 [31, 37, 38]).

Results from the NMAs (Table 2) showed no statistically significant differences between Enco + Bini and Dab + Tram for the outcomes of investigator-assessed PFS and OS. Three different HRQoL NMA results were estimated: pre-progression, difference in change from baseline at week 32, and at disease progression. The HRQoL results all favoured Enco + Bini (Delta < 0); however, the credible intervals (CrIs) cross 0 for all analyses, therefore the differences were not statistically significant.

**Table 2:** PFS, OS, AEs and HRQoL from the NMA produced by the company

Outcome	Enco + Bini vs Dab + Tram
PFS (investigator assessed)	
HR (95% CI)	0.77 (0.57–1.04)
OS	
HR (95% CI)	0.89 (0.65–1.23)
AEs	
Any grade ≥ 3 AEs	
OR (95% CI)	1.18 (0.70–1.98)
Any serious AEs	
OR (95% CI)	0.86 (0.52–1.43)
HRQoL	
EQ-5D utility score, pre-progression	
Dt (95% CI)	−0.02 (−0.05 to 0.01)
EQ-5D utility score, DCFB at week 32	
Dt (95% CI)	−0.04 (−0.10 to 0.02)
EQ-5D utility score, DCFB at disease progression	
Dt (95% CI)	−0.04 (−0.12 to 0.04)

*AE* adverse events, *Bini* binimetinib, *CrI* credible interval, *Dab* dabrafenib, *DCFB* difference in change from baseline, *Dt* delta, *Enco* encorafenib, *EQ*-*5D* EuroQol-5 dimensions, *HR* hazard ratio, *HRQoL* health-related quality of life, *NMA* network meta-analysis, *OR* odds ratio, *OS* overall survival, *PFS* progression-free survival, *Tram* trametinib

NMA results for the incidence of any grade ≥ 3 AEs favoured Dab + Tram (odds ratio [OR] > 1), while results for serious AEs favoured Enco + Bini (OR < 1). However, for both analyses, the CrIs crossed 1, meaning a statistically significant difference was not demonstrated.

#### Critique of Clinical Evidence and Interpretation

The ERG was satisfied with the company’s literature search strategy approach and was confident that the search was carried out to an acceptable standard. The ERG did not identify any additional studies that should have been included in the company’s review.

The ERG considered that the COLUMBUS trial [21–23] was of good quality and was well-conducted, with blinded independent review of PFS outcomes and collection of HRQoL data. The ERG noted that the patients recruited to the trial were largely representative of patients with advanced (unresectable or metastatic) BRAF V600 mutation-positive melanoma who are likely to be treated in the NHS, with the caveat that very few patients in the COLUMBUS trial [21–23] had brain metastases and none of the patients had a poor performance status (PS) (i.e., PS ≥ 2).

The results from the COLUMBUS trial [21–23] did not provide evidence for the clinical effectiveness of Enco + Bini versus Dab + Tram, the comparator specified in the final scope issued by NICE.

The ERG interpreted the results of the company’s NMAs with caution due to several issues relating to the methods, including the sparsity of evidence in the networks (particularly the HRQoL network), the variability in the lengths of trial follow-up (2–6 years), differences between trials in median follow-up for OS (11–33.3 months), the inclusion of dacarbazine within the networks (which is not a BRAFi and is no longer regarded as a standard NHS treatment), and the fact that only an NMA of PFS by local investigator review (rather than BIRC) was feasible. Five of the seven trials (COLUMBUS [21–23], COMBI-v [24, 25], BRF113220 Part C [29–33], BREAK-3 [31, 37, 38] and BRIM-3 [39–42]) included within the NMAs were designed as open-label studies; the ERG noted that investigator assessment of PFS in open-label trials may be subject to bias.

The ERG also noted that, in the final scope [20], NICE did not consider immunotherapies to be appropriate comparators to Enco + Bini. However, clinical advice to the ERG was that, in the NHS, many patients with advanced (unresectable or metastatic) BRAF V600 mutation-positive melanoma are treated with a programmed cell death protein 1 (PD-1) inhibitor immunotherapy as a first-line treatment and the rationale for not including immunotherapies as comparators in the final scope was unclear to the ERG. The ERG considered that, as many NHS patients are offered an immunotherapy as a first-line treatment, results from the company’s NMAs are only relevant to patients in the NHS who are likely to be treated with targeted therapies, i.e. patients with highly symptomatic or rapidly deteriorating disease.

### Cost-Effectiveness Evidence

#### Overview of Company’s Economic Evidence

The company’s economic evaluation compared the cost-effectiveness of Enco + Bini versus Dab + Tram when used to treat advanced (unresectable or metastatic) BRAF V600 mutation-positive melanoma. Using Microsoft Excel, the company produced a partitioned survival model with a 30-year time horizon and monthly cycles. The UK NHS was the perspective of the model, in line with the NICE reference case [1]. Outcomes were measured in quality-adjusted life years (QALYs), and both costs and QALYs were discounted at an annual rate of 3.5%, as recommended by NICE [1]. The model comprised three mutually exclusive health states: progression-free (PF), post-progression (PP) and death. Sub-states within the PF and PP health states were included to take into account whether patients were on or off primary treatment. The survival curves representing the experience of patients treated with Enco + Bini in the COLUMBUS trial [21–23] are used for Enco + Bini in the economic model, with parametric models fitted to the COLUMBUS trial [21–23] data in order to extrapolate beyond the time horizon of the trial. In the absence of direct evidence comparing Enco + Bini versus Dab + Tram, the survival curves for Enco + Bini were adjusted using HRs generated by the company’s NMAs to obtain survival curves for Dab + Tram. The company assumed that the time on treatment was the same for patients receiving Enco + Bini and Dab + Tram, and used primary time on treatment data for both treatment combinations from the Enco + Bini arm of the COLUMBUS trial [21–23]. Different relative dose intensity (RDI) multipliers and grade 3/4 AEs incidence rates occurring in ≥ 5% of patients were used in the company economic model for Enco + Bini (using data from the COLUMBUS trial [21–23]) and Dab + Tram (using data from the COMBI-v trial [24, 25] and COMBI-d [26–28] trial). Utility values for the PF and PP health states were derived from the company’s NMA. The PF on-treatment utility values differed by primary treatment, whilst the same utility values were used, regardless of primary treatment for PF off-treatment and PP. Resource use and costs were estimated based on information from the COLUMBUS trial [21–23] (usage of primary and subsequent treatments, and AE rates), published sources—including an Australian study of people with melanoma to estimate health state resource use [43], with the unit costs of the resource use based on estimates from the NHS [44, 45], estimates of AE costs [46] and terminal care costs [47]—and advice from experts in clinical practice in the NHS.

Confidential Patient Access Scheme (PAS) prices, discounted prices for the NHS, agreed with the Department of Health, were in place for Enco + Bini and for Dab + Tram at the time of this appraisal. As the company was unaware of the PAS prices for the comparator treatments, the company base-case results only included the PAS price for Enco + Bini. Using this reduced price, the results from the company’s economic model estimate that Enco + Bini dominates Dab + Tram, generating 0.453 additional QALYs at a reduced cost.

The company carried out probabilistic and deterministic sensitivity analyses. The results showed that the base-case analyses results were sensitive to the use of an estimated HR for time to treatment discontinuation (TTD) (in comparison to making TTD equal for Enco + Bini and Dab + Tram in the base case) and the dose of Dab + Tram (drug dose each time it was administered and RDI). Two scenarios in which Enco + Bini did not dominate Dab + Tram were produced by the company; one of which included discounts to the list prices of dabrafenib and trametinib, and the other assumed Enco + Bini and Dab + Tram were equally effective and safe (OS, PFS, utility values in the PF state and AE rates).

#### Critique of Company’s Cost-Effectiveness Evidence

The ERG considered that the design of the company model was appropriate and that it was generally well structured. In addition, the ERG was satisfied with the way in which the COLUMBUS trial [21–23] data were incorporated into the model.

The COLUMBUS trial [21–23] data were used to populate the company economic model; OS, PFS, time on treatment data, utility values and AE rates were used for Enco + Bini. The company used the numerically, but not statistically significant, results from their NMAs within the economic model to estimate OS, PFS and utility values for Dab + Tram. The ERG, however, considered it inappropriate to model any differences between the treatments for these outcomes as the numerical differences were not statistically significant. The company used the incidence rates of grade 3 and 4 AEs occurring in at least 5% of the patients from the COLUMBUS [21–23], COMBI-v [24, 25] and COMBI-d [26–28] trials, rather than the results of the NMAs. The ERG considered that this was not a robust approach as it failed to account for any differences in patient baseline characteristics across the three trials.

Based on the lack of any evidence supporting a demonstrable difference in outcomes between Enco + Bini and Dab + Tram, the ERG considered that the only parameters that could affect model results were treatment-related costs. In the company model, these were a function of time on treatment, administration costs, RDI and drug costs. The ERG was satisfied that the time on treatment estimates and the administration costs (given the same mode of delivery) for Enco + Bini and Dab + Tram were the same, as assumed in the company base case. The company employed different RDI multipliers in their estimation of treatment costs for Enco + Bini and Dab + Tram. However, as the ERG considered that there was no robust evidence to support differential AE estimates for each of the treatment options, there was equally no robust evidence to support the use of different RDI multipliers.

The ERG’s opinion was that equivalence of time on treatment, drug administration costs and RDI across treatment options meant that drug prices were the only cost difference to consider; therefore, to establish cost-effectiveness, a simple cost-minimisation analysis, rather than a cost-utility analysis, was appropriate.

### Conclusions of the ERG Report

The objective of this appraisal was to compare the clinical and cost-effectiveness of Enco + Bini versus Dab + Tram for adults with advanced (unresectable or metastatic) BRAF V600 mutation-positive melanoma. As Dab + Tram was not a comparator in the COLUMBUS trial [21–23], the company carried out a series of NMAs to compare Enco + Bini versus Dab + Tram in terms of efficacy (PFS and OS), safety outcomes and HRQoL. The results of these NMAs showed that there was no statistically significant difference between the two treatments for any of these four outcome measures. The ERG was satisfied that there was no robust evidence of any statistically significant clinical differences between OS or PFS outcomes, utility values or AE profiles when Enco + Bini was compared with Dab + Tram. The ERG considered that the only difference between the two treatment combinations that affected model results was treatment-related costs; a cost-minimisation analysis was therefore an appropriate approach for comparing the cost-effectiveness of the two treatments. Using the ERG’s preferred scenario (equivalent OS, PFS, utility values, AEs and RDI multipliers) and PAS prices for Enco + Bini, Enco + Bini was less costly than Dab + Tram, and as estimated total QALYs were also assumed to be equal, the ERG considered that Enco + Bini was a cost-effective alternative to Dab + Tram.

### Methodological Issues

The lack of direct comparative evidence between Enco + Bini and Dab + Tram resulted in the company carrying out NMAs to provide estimates for each of the key outcomes. The networks did not, however, generate estimates of effects for Enco + Bini that statistically significantly differed from estimates for Dab + Tram. The ERG considered that estimating differences between Enco + Bini versus Dab + Tram based on estimates from the NMAs in the economic model was inappropriate and, therefore, carried out a cost-minimisation analysis.

As the company assumed time on treatment to be the same for Enco + Bini and Dab + Tram, and in the ERG’s scenarios the key outcomes (OS, PFS, HRQoL and AEs) were assumed to be the same for both treatment options, the ERG considered that the proposed difference in RDI in the company model was difficult to justify. The ERG adjusted the model so that the RDI was the same for the two comparator treatments.

## NICE Guidance

### Clinical Need

The AC heard from a patient and clinical experts that having the choice of two targeted combination treatments would improve patients’ HRQoL, particularly given that Enco + Bini and Dab + Tram have different toxicity profiles. The AC concluded that both clinicians and patients would welcome an additional treatment option.

### Current Management

The AC heard from clinical experts that the treatment of patients with unresectable or metastatic BRAF V600 mutation-positive melanoma is evolving, with new immunotherapy and other treatments becoming available. There is also variation in practice across the NHS as to the use of targeted therapies or immunotherapies, alone or in combination, as first-line therapy; however, clinical experts agree that most patients will receive targeted therapies at some time. The clinical experts discussed a preference for the use of immunotherapies in the first-line setting for patients with a good PS, but stated that this was not a practice adopted throughout the NHS. The AC noted that the position of targeted therapy in the treatment pathway was not easy to establish.

### Clinical Effectiveness

The AC heard from the ERG that the population in the COLUMBUS trial [21–23] had a good PS and few had brain metastases. The clinical experts explained that having a generally good PS even with advanced disease is common in people with unresectable or metastatic BRAF V600 mutation-positive melanoma.

Although the ERG raised a potential concern that the proportion of patients receiving immunotherapies as a first-line treatment in the COLUMBUS trial [21–23] was low (6%), the AC did not consider this to be a major limitation as there is no consensus in NHS practice as to whether immunotherapies or targeted therapies, should be used to treat treatment naïve patients with advanced mutation-positive melanoma. The clinical experts advised that treatment with immunotherapies in the first-line setting are unlikely to affect treatment response to targeted therapies used later in the treatment pathway as their modes of action are different.

The AC considered the COLUMBUS trial [21–23] to be well conducted and to represent clinical practice in the NHS in England. However, the AC noted that the COLUMBUS trial [21–23] did not directly compare Enco + Bini with the comparator in the NICE scope, Dab + Tram.

The AC noted the lack of statistically significant differences in PFS, OS, AEs or HRQoL demonstrated in the company’s NMAs. The AC considered that the results of the NMAs should be viewed with caution due to their wide CrIs in all base-case and sensitivity analyses and the limitations highlighted by the company and ERG in terms of the construction of the networks. The clinical experts indicated that Enco + Bini and Dab + Tram are likely to have similar clinical effectiveness estimates. The AC concluded that clinical effectiveness estimates are likely to be similar, but are associated with uncertainty.

The AC considered the rates of AEs within the COLUMBUS trial [21–23] to be similar for Enco + Bini and vemurafenib, which were, in general, infrequent and with few serious events reported. Although the COLUMBUS trial [21–23] does not offer a direct comparison with Dab + Tram, the AC heard from clinical experts that patients treated with Dab + Tram sometimes develop pyrexia, which can lead to reduced treatment dosage or hospitalisation. Pyrexia appears to occur less frequently with Enco + Bini than with vemurafenib in the COLUMBUS trial [21–23], and therefore the AC considered that the safety profile of Enco + Bini may be more favourable than that of Dab + Tram.

### Cost-Effectiveness

The AC was satisfied that the company’s model was appropriately structured for decision-making. The AC accepted the ERG’s amendment to RDI to Enco + Bini, which brought it in line with that of Dab + Tram, justified by the fact that the comparator combination has the same mode of delivery.

The AC noted the reliance of the results of an economic model on an indirect comparison with Dab + Tram for the comparative clinical effectiveness estimates of Enco + Bini. The company’s model included the outputs of the NMAs, which included small numerical differences in estimates of PFS, OS, AEs and HRQoL in Enco + Bini versus Dab + Tram. The ERG suggested a cost-minimisation approach to the analysis, assuming clinical equivalence, as the company’s NMA did not demonstrate any significant differences in investigator-assessed PFS, OS, AEs or HRQoL between the combination treatments Enco + Bini and Dab + Tram. The clinical experts advised the AC that similar efficacy between the two combination treatments is biologically plausible. The AC was concerned that the absence of evidence does not equate to evidence of absence, and therefore clinical equivalence is not proven by the company’s indirect comparisons. Given that the numerical differences (from the company’s NMA results) included in the company economic model were small, the AC concluded that both the assumptions made in the company base-case analysis of a difference in effectiveness between Enco + Bini and Dab + Tram and the ERG’s cost-minimisation approach were appropriate for decision-making.

The AC were presented with the economic analyses results using confidential commercial arrangements for all four drugs in the treatment combinations Enco + Bini and Dab + Tram.

### Final Guidance

The AC recommended Enco + Bini for the treatment of adult patients with unresectable or metastatic melanoma with a BRAF V600 mutation. The final guidance was published by NICE in February 2019 [2].

## Conclusion

There is no direct evidence to confirm any differences in clinical effectiveness between Enco + Bini, and Dab + Tram and the available indirect evidence is unreliable. As a result, the AC agreed that a cost-minimisation approach was an appropriate method to compare the treatment options.

There is no evidence of the clinical and cost-effectiveness of Enco + Bini versus immunotherapies, such as nivolumab and pembrolizumab. Immunotherapies are recommended for use in all patients, regardless of mutation status; however, there is no consensus as to the optimal approach to the sequencing of BRAF-targeted therapies and immunotherapies. Clinical advice to the AC was that the outcomes for patients would be similar regardless of treatment sequencing. Comparisons between Enco + Bini and immunotherapies were not the focus of this appraisal.

Commercial access arrangements are in place for both treatment options, and therefore the incremental cost-effectiveness ratios per QALY gained are confidential; however, the AC was satisfied that Enco + Bini was likely to be a cost-effective use of NHS resources and therefore recommended its use in patients with BRAF V600 mutation-positive unresectable or metastatic melanoma.
